# Vaccination against influenza in pregnant women in a maternity hospital in the Czech Republic in the season 2020–2021

**DOI:** 10.1186/s12889-023-15911-5

**Published:** 2023-05-31

**Authors:** Jan Kynčl, Monika Liptáková, Jana Košťálová, Marek Malý, Marcela Křížová, Hynek Heřman, Kateřina Fabiánová

**Affiliations:** 1 Centre for Epidemiology and Microbiology, Department of Infectious Diseases Epidemiology, National Institute of Public Health, Prague, Czech Republic; 2 Third Faculty of Medicine, Department of Epidemiology and Biostatistics, Charles University, Prague, Czech Republic; 3 Department of Biostatistics, National Institute of Public Health, Prague, Czech Republic; 4 Institute for the Care of Mother and Child, Prague, Czech Republic; 5 Third Faculty of Medicine, Charles University, Prague, Czech Republic

**Keywords:** Influenza, Pregnancy, Vaccination, Health knowledge, Prevention

## Abstract

**Objectives:**

Pregnant women are among the priority groups to receive influenza vaccines in the Czech Republic since 2011, data on vaccination coverage are not yet available. The aim of the study was to determine the influenza vaccination coverage (IVC) and provide source data for further activities.

**Methods:**

A prospective observational study was performed in a large maternity hospital in Prague. The self-completed questionnaire was distributed to 5,475 pregnant women between September 1, 2020 and August 31, 2021. Questions included maternal sociodemographic characteristics, influenza vaccination status and sources of maternal vaccination recommendations during pregnancy.

**Results:**

A total of 4,617 completed questionnaires have been analysed. The median age of study participants (N = 4,592) was 33 years (range: 18–51 years). The majority (69.7%) of women had completed their university education, most women were childless (58.5%) or had one child (32.5%) before the start of the study. Less than 2% of women reported being vaccinated against influenza during their pregnancy (1.5%; 95% CI, 1.1–1.9%). Only 21% of women knew that it’s possible to get vaccinated against influenza during pregnancy. Participants considered influenza vaccination in pregnancy as important (3.3%), useful (41.1%) and useless (44.4%). Out of 959 pregnant women who had information about influenza vaccination during pregnancy, only 6.9% were vaccinated, while among those who did not have this information, 0.1% were vaccinated during pregnancy (p < 0.001). The most frequent source of information was Internet, then media and a general practitioner.

**Conclusions:**

The IVC during pregnancy in our study was extremely low. In order to improve IVC among pregnant women, it is necessary to increase awareness of recommendations and vaccination options among the public and professionals and incorporating vaccination recommendation in routine antenatal practice.

**Supplementary Information:**

The online version contains supplementary material available at 10.1186/s12889-023-15911-5.

## Introduction

Seasonal influenza is an acute viral infection that occurs worldwide with seasonal epidemics and is estimated to cause 3–5 million severe cases and up to 650 000 respiratory deaths every year [1, 2].

Course of the disease can be severe among the elderly, people with chronic diseases and other conditions [3] including pregnant women. The influenza in pregnancy can cause several complications [3, 4]. Pregnant women have an increased risk of serious respiratory illnesses and hospital admissions during influenza season [5]. Also the newborn babies and infants are in the risk of influenza complications, namely hospitalization and death [6].

The World Health Organization (WHO) recommendation for influenza vaccination of pregnant women has been in place since 2005 [3, 5] and has been launched over time in most European countries [5, 7]. Pregnant women and their newborn babies have a high likelihood of severe disease; therefore, the WHO has included them as a priority group for influenza vaccination since 2012. The prevailing recommendation is universal (to all women at any trimester of pregnancy), but in some countries the recommendation covers only pregnant women with high risk condition or in the second and third trimesters of pregnancy [3, 8]. Despite very good safety profile of influenza vaccines, influenza vaccination coverage in pregnant women in many countries remains low [5].

It is known, that vaccination provides direct and indirect benefits to pregnant women, including reduced morbidity in their newborns and infants up to 2–3 months of age [9]. Seasonal influenza vaccination is specific because of the relatively short time interval for vaccination. The appropriate timing for influenza vaccination in pregnancy mainly includes influenza season and trimester of pregnancy [9].

Although vaccination against influenza, which also includes pregnant women, is recommended by the National Immunization Committee in the Czech Republic (Czechia) already since 2011 and by the Czech Vaccination Society of the Czech Medical Association [10, 11], data on the vaccination coverage of pregnant women are not yet available. In Czechia, influenza vaccination is recommended for pregnant women at any trimester of pregnancy and women who plan to become pregnant during the influenza season. However, the vaccine and administration are not covered by health insurance and must be paid for by the vaccinated person. As influenza vaccine recommendations are not included in prenatal guidelines in the Czech Republic at the moment, the professional society of gynaecologists in the Czech Republic has decided to support the recommendation - European Board and College of Obstetrics and Gynaecology position statement: vaccination in pregnancy [12]. Influenza vaccine coverage (IVC) in the ≥ 65 years age group is consistently low in Czechia: 24.0% in the 2006/2007 season and 22.4% in the 2020/2021 season [13].

The aim of this study was to determine the IVC in a population of pregnant women and to analyse obtained information in order to identify potential barriers related to vaccination against influenza.

## Methods

Prospective observational hospital-based study in the maternity ward of the Institute for Mother and Child Care (UPMD), with an approximate birth rate of 5,300 births/year, located in Prague was performed.

In Czechia, pregnant women can get an influenza shot for a fee directly from their GP or an antenatal care provider, both public and private. As part of its benefits, health insurance companies (HICs) offer their insured persons reimbursement of influenza vaccinations not covered by public health insurance, based on a completed application and proof of payment.

Potential participants were informed about the aim and course of the study through informed consent. They were asked if they agreed to participate in the study, and also asked to sign an informed consent and fill out a questionnaire. Health care workers (HCWs) provided both documents to the potential participants in the survey in UPMD. The questionnaire was distributed to pregnant women in antenatal outpatient care, upon admission to the maternity hospital for given birth, or before leaving the maternity hospital UPMD. All participants were asked to complete the questionnaire regarding their experiences and history of vaccination against influenza from 1st September 2020 to 31st August 2021.

The questionnaire (Supplementary Table S1) and informed consent were available in Czech or English language to include as many patients as possible. All participants were Czech residents or foreigners living in Czechia and giving birth in UPMD.

All available self-completed paper-based questionnaires were transferred to the National Institute of Public Health (NIPH) for data entry and analysis.

Data analysis concerns answers from all completed questionnaires. The patient survey contained questions on demographics (date of birth, education, number of children) and vaccination-related questions (opinion about influenza vaccine in pregnancy and data about influenza vaccination including administration in pregnancy). To assess the knowledge of pregnant women regarding influenza, they were asked if they were aware of information to be vaccinated during pregnancy. In case they were aware, their information source was asked for.

Collected data were analysed anonymously. The results are presented as proportions and percentages of respondents to individual questions, excluding non-responses from the denominators. Mean or median were estimated for continuous variables. Fisher’s exact test was performed to analyse the associations between categorical variables. Estimate of IVC was calculated with 95% confidence interval (CI). Findings were reported as statistically significant at p < 0.05. Statistical analyses were performed in STATA version 17 (StataCorp LLC, College Station, Texas, USA).

## Results

Of the 5,475 women who gave birth in UPMD during the one-year study period, 4,617 (84%) completed the questionnaire and were included in the analysis. The participants were mainly from Prague and the surrounding area, but the place of residence was not part of the survey.

The median age of study participants (N = 4,592) at the end of the study was 33 years (range: 18–51 years, interquartile range 6); the most represented age group was 30–34 years (42%) (Table 1). Most of the participants completed university education (69.7%) and 58.5% were childless before the start of the study (Table 1).

**Table 1:** Demographic characteristics of surveyed pregnant women in the Czech Republic

Demographic characteristics N = total number of completed surveys	Number of pregnant women	Percent of pregnant women (%)
**Age group (years), N = 4,592**		
18–24	147	3.2
25–29	941	20.5
30–34	1,919	41.8
35–39	1,248	27.2
40–44	308	6.7
45–51	29	0.6
**Education level (N = 4,617)**		
University	3,218	69.7
Secondary school	1,316	28.5
Primary school	83	1.8
**Number of children (N = 4,617)**		
0	2,702	58.5
1	1,500	32.5
2	353	7.7
3	49	1.0
4	9	0.2
5	4	0.1

Self-reported IVC during pregnancy was 1.5% (95% CI, 1.1-1.9%). Of the 68 women who were vaccinated against influenza in pregnancy, 66 (97.1%) reported time of the vaccination, which was mostly in the second or third trimester (Table 2). The proportion of vaccinated women was higher among women with university education (1.7%) than among women with primary or secondary education (0.9%), p = 0.046. The proportion of vaccinated women increased significantly (p = 0.002) with age (0.6%, 1.4%, 2.2% and 2.5% respectively in the age groups 18–29, 30–34, 35–39, 40–51).

Of the 677 women who had ever received influenza vaccine, 668 reported how often they had been vaccinated, with just under 13% receiving the shot regularly every year.

The opinion that vaccination against influenza during pregnancy is useless was held by 44.4% of study participants, and a similar proportion (41.1%) of the women were convinced of the usefulness of vaccination.

Only about 21% of all participants (959/4616) were aware of the possibility to be vaccinated against influenza during pregnancy (Table 2). The most common source of information about influenza vaccination in pregnancy was the Internet, followed by other media, a general practitioner (GP) and a gynaecologist. Several pregnant women mentioned in the questionnaire that their physician does not recommend vaccination against influenza in pregnancy.

**Table 2:** Influenza vaccination characteristics of surveyed pregnant women in the Czech Republic

Vaccination characteristics N = total number of completed surveys	Number of pregnant women	Percent of pregnant women (%), in respective subgroup
**In your opinion, influenza vaccine in pregnancy (N = 4,617)**		
Is important	152	3.3
Is useful	1,895	41.1
Is useless	2,050	44.4
Should be prohibited	228	4.9
I do not know	292	6.3
**Have you ever received influenza vaccine? (N = 4,614)**		
Yes	677	14.7
No	3,937	85.3
**How often do you get vaccinated against influenza? (N = 668)**		
Every year	85	12.7
Sometimes	583	87.3
**Have you received influenza vaccine during pregnancy? (N = 4,617)**		
Yes	68	1.5
No	4,549	98.5
**In which trimester of pregnancy did you receive influenza vaccine? (N = 66)**		
First trimester	11	16.7
Second trimester	32	48.5
Third trimester	23	34.8
**Were you aware of the possibility to get vaccinated against influenza during pregnancy? (N = 4,616)**		
Yes	959	20.8
No	3,657	79.2
**Source of information regarding vaccination against influenza during pregnancy (N = 959)** *		
Internet	343	35.8
Media	172	17.9
General practitioner	152	15.8
Gynaecologist	96	10.0
Family	78	8.1
Friend	45	4.7
Other	198	20.6

Note: *Multiple answers were allowed in this question, so the total exceeds 100%

Of 959 pregnant women who were informed about influenza vaccination during pregnancy, only 6.9% (66/959) were vaccinated, while among those who were not informed, even only 0.1% (2/3,657) were vaccinated during pregnancy (Fisher’s exact test, p < 0.001).

## Discussion

To the best of our knowledge, this study is the first single study-centre survey analysing IVC among pregnant women in Czechia.

UPMD is one of the largest maternity hospitals in Czechia. In 2021, there were 5,598 births (5,761 single children, 159 twins and 1 triplet); in 2020, there were 5,164 births (5,330 single children and 166 twins [14]. A total of 111.8 thousand children were born alive during 2021 in Czechia, 1.6 thousand more than in 2020 [15]. Therefore, our study represents a group of 5.2% of all children born in Czechia.

The study was performed during the crisis of the health-care system caused by the COVID-19 pandemic. Nevertheless, in the first year of the COVID-19 pandemic (i.e. during the 2020/2021 season) influenza vaccination in Czechia was promoted as a central public health measure based on limited evidence that it can greatly benefit the management of the coronavirus pandemic, e.g. facilitating differential diagnosis and avoiding an overload of health services and hospitals associated with influenza infections [16]. There was no decrease in IVC in Czechia in that season and all available influenza vaccines were completely used.

In the Czech study by Riad et al., only 10 (2.8%) participants reported receiving vaccines other than the COVID-19 vaccine during pregnancy, including influenza and diphtheria, tetanus, and pertussis (DTP) vaccines [17].

In the online and telephone survey among GPs from ten EU member states, 6.5% of 107 interviewed Czech GPs did not believe that influenza vaccination was important, and 1.9% of them did not consider it as safe. Nevertheless only 25.2% of them would recommend the influenza vaccination to pregnant women [18].

The average age of women giving birth in Czechia in 2020 was 30 years [19], which is 3 years less than among participants of our study. The peak of fertility also shifted over time to an older age of 30–34 years [19], which corresponds to the age distribution of our study participants. In the period between 2011 and 2018, the total fertility increased from 1.43 children per woman to 1.71 children [19].

University education was highly overrepresented in our study (69.7%), as the share of university education among women of childbearing age 15–49 in Prague is 42.4% according to the Czech Statistical Office (CZSO) [20]. We do not have a clear and single explanation for this result. UPMD is a highly specialized facility that provides health care in gynaecology and obstetrics mainly for the catchment area of Prague and the Central Bohemia Region. Therefore, we consider it as the main reason why participants of the study had higher education.

The structure of the study population, in terms of the number of children, corresponds general data for Prague.

Although the influenza vaccination has been recommended in Czechia since 2011, the IVC among pregnant women in our study was extremely low (1.5%). Similarly low results were reported in a ten-year analysis from a French healthcare database, where only 1.2% pregnant women were vaccinated against influenza in the 2009–2018 period (N = 875/72,207; 95% CI 1.14–1.30) [2]. The IVC among pregnant women slightly increased in France after the 2012 WHO recommendation, from 0.33 to 1.79% (p < 0.001) but even in later times, it is still very low (4.1% in 2018) [2]. The estimated IVC among pregnant women in China in 2004–2014 was < 1.5%, which is consistent with other published Chinese regional estimates [21].

In 2008–2009, 40% of the European Union countries implemented recommendations to vaccinate pregnant women, compared to 91% in 2014–2015 [8, 22]. Based on data from the European Centre for Disease prevention and Control, the lowest IVC among pregnant women in 2016–2017 was 0.5% in Slovenia and the highest 58.6% in the United Kingdom (UK) (median 25.0%) [2, 23]. Also in neighbouring Germany IVC among pregnant women increased from 9.0 to 16.6% between seasons 2014/15 and 2019/20 based on national outpatient claims data [24]. In the United States of America (USA), the percentage of pregnant women who received influenza vaccination increased from 49.1% during the 2017–18 influenza season [25] to 61.2% during the 2019–20 influenza season [5, 26]. A large New Zealand study showed that IVC among pregnant women nearly tripled from 11.2% to 2013 to 30.8% in 2018 [27]. In Spain, the IVC among pregnant women increased significantly during latest decade and reached 50% [28]. In Irish study, the IVC was 61.7% among 241 pregnant women in 2017–18 influenza season [29]. The self-reported IVC among pregnant women for the 2018–19 season in Italy was 15% (72/483) and 6.5%, during the 2017–18 influenza season [30].

In our study, most of the pregnant women were not aware of the possibility to get vaccinated against influenza in pregnancy. In few cases, women in our study mentioned in the questionnaire that the physician recommended not to vaccinate during pregnancy.

Irish study identified that women with higher socio-economic background, those with a university degree and those who attended as a private or semiprivate patient were more likely to get vaccinated against influenza in pregnancy [5, 31].

A recent multi-centre questionnaire study from the UK investigated the acceptability of antenatal vaccination among patients and the HCWs level of confidence in vaccine recommendations [5]. This study found that the most commonly cited reason for declining antenatal vaccination was concerns regarding possible side effects for the baby and doubts regarding efficacy and necessity of immunisation [5]. Immunisation rates are thought to be higher when HCWs recommend, offer, and administer the vaccine at the same visit, then in a situation where they make a recommendation and refer the patient elsewhere to receive the vaccine [5].

Health care setting was the most cited source of information (87.5%) followed by TV (18.4%) in Irish study [29]. The most common reasons for receiving influenza vaccine were: GP recommendation (39.2%), and being pregnant (23.8%) in Irish study [29]. Results from Irish study are considerably different in comparison with our study in which the role of HCWs was not an essential source of information for the pregnant women.

Psarris et al. led a study in Greece in 2018 where the proposition rate rose from 27 to 100% and the IVC from 14 to 94% after a simple information campaign towards healthcare professionals [2, 32].

An increasing number of countries have issued recommendations for the use of influenza vaccines during pregnancy, and are offering these vaccines free of charge. However, even in such countries, despite the demonstrated effectiveness and strong safety profile of maternal influenza vaccination, IVC has remained suboptimal [33].

We recommend raising the awareness of pregnant women about the fact that there are contributions from HICs for vaccinations that they can use. We agree with the Czech Vaccination Society’s recommendation that HICs provide full influenza vaccination reimbursement to all pregnant women to ensure maximal IVC [11]. Most women (80.6%) reported to be aware of the influenza vaccine campaign, and this group was more likely to get vaccinated according to Irish study [29].

Based on a recent systematic review concerning determinants of influenza vaccine hesitancy among pregnant women in Europe, there were no studies from Eastern Europe [34]. However, it should be noted that the vaccine hesitancy is present also among HCWs. A comparative study in six European countries including the Czech Republic demonstrated that 27% of the Czech HCWs (mainly GPs) showed hesitant sentiment for influenza vaccination acceptance [35].

The latest survey on the attitude of Czech society towards influenza and influenza vaccination showed that 22% of respondents planned to get vaccinated against the influenza in the 2022/2023 season. Of the previously unvaccinated, only less than 3% were considering vaccination. Unvaccinated people stated that they do not get the influenza, that they do not trust vaccinations, and that they do not belong to the risk groups [36].

To help increasing the coverage, gynaecologists or GPs should actively recommend maternal influenza vaccination to their patients during the first antenatal visit. They should give the information about the effectiveness and safety of maternal immunisation, and about the risk of infection and severity of the respective diseases in the absence of vaccination. Ideally, vaccines should be offered on-site during one of the routine antenatal visits, thereby maximizing convenience for the patient [33].

Results of a multi-Centre survey study in Italy showed that receiving a HCWs vaccine advice and the availability of vaccines during prenatal care visits might improve vaccination coverage among pregnant women [30]. The lack of HCW vaccine recommendation was identified as the most important vaccination barrier among pregnant women [30, 36–39].

The combination of HCW recommendation and educational materials on the vaccines was significantly predictive of influenza vaccine acceptance [38]. In the USA study, although all providers reported recommending the influenza vaccines in pregnancy, about 30% of women did not recall receiving a recommendation [38].

Recommendation by a HCW was identified as the strongest predictor of antenatal vaccination [40]. GP recommendation was the main reason for receiving influenza vaccine (39.2%) in a cross-sectional survey in 2017–2018 influenza season performed among pregnant women in Ireland [29].

To get an overview of real IVC, it would be useful to conduct a study in order to assess the IVC among pregnant women based on the data available in the new nationwide electronic vaccination register launched in Czechia in January 2022. Other proposal is to perform a survey in Czechia whether HCWs provide recommendation for vaccination in pregnancy. Measures such as studies of the knowledge, attitudes and practices of both pregnant women and HCWs need to be undertaken to improve the coverage [2].

At the moment, activities already conducted in the Czech Republic to increase IVC among pregnant women includes presentations for HCWs at scientific conferences and publication of papers for improving knowledge [41]. We believe that provisioning of educational materials (e.g. leaflets, posters) for vaccination in pregnancy and distributing them especially in waiting rooms of outpatient department of gynaecologists and GPs could raise awareness and help to improve IVC in pregnancy. These materials should be supported by the key stakeholders, including the Ministry of Health of the Czech Republic.

A strength of this study was the high participation rate (84%) and the study size (4,617 participants). Limitations of this study are related to the nature of an observational study. The use of a self-completed questionnaire enabled us to limit the potential for recall bias. This single-centre study could have induced recruitment bias as participants were enrolled from a large specialized hospital, which provides health care primarily to patients from Prague. Therefore, our study population is not a representative sample for estimation of IVC of pregnant women in whole Czechia. Future studies should include a wider geographical area and different levels of hospitals to provide a better overview of the current situation. Vaccination status was reported by the women and therefore we can expect reporting bias, which could not be verified due to the lack of a national vaccination register.

## Conclusions

Although the WHO has recommended influenza vaccination for all pregnant women since 2012, we found extremely low IVC in our study. Therefore, it is necessary to increase awareness about recommendation on vaccination in pregnancy among HCWs and public and to incorporate it into routine antenatal care. In addition, we think that free of charge vaccination, or at least higher reimbursement from HICs could improve the IVC among pregnant women in Czechia. Measures need to be undertaken to improve the IVC, such as national information campaigns about influenza vaccination (more than the current annual press conference), disseminating information about the vaccination in pregnancy, and engaging HCWs during professional conferences and via medical scientific societies. It seems useful to perform a study of IVC among pregnant women based on data from a new nationwide electronic vaccination register in the future.

## Electronic supplementary material

Below is the link to the electronic supplementary material.

Supplementary Material 1

## Data Availability

The dataset used and analysed during the study is available from the corresponding author on reasonable request.
