# Supplementary material for: Vaccination against influenza in pregnant women in a maternity hospital in the Czech Republic in the season 2020–2021
Source: BMC Public Health. 2023 May 31;23:1029. doi: 10.1186/s12889-023-15911-5 (PMC10230122; doi:10.1186/s12889-023-15911-5)
Supplement: Supplementary file 1 — Supplementary Material 1 [file 12889_2023_15911_MOESM1_ESM.docx]

**Supplementary file**

**Vaccination against influenza in pregnant women in a maternity hospital in the Czech Republic in the season 2020–2021**

**Supplementary Table S1** Patient survey questions regarding influenza vaccination in pregnancy

| **1. What is the highest level of education you have completed?** | |
| --- | --- |
| Primary | Secondary |
| University |  |
| **2. How many children have you got?** | |
| **3. In your opinion, influenza vaccine in pregnancy** (please tick one option): | |
| Should be prohibited | Is useless |
| Is useful | Is important |
| **4. Have you ever received influenza vaccine?** | |
| Yes | No |
| **4a. If so, please indicate how often you get vaccinated against influenza:** | |
| Every year | Sometimes |
| **5. Have you received influenza vaccine in pregnancy?** | |
| Yes | No |
| **5a. If so, please indicate in which trimester of pregnancy:** | |
| First trimester (months 1-3) | Second trimester (months 4-6) |
| Third trimester (months 7-9) |  |
| **6. Were you aware of the possibility to get vaccinated against influenza in pregnancy?** | |
| Yes | No |
| **6a.** **If so, please indicate who you got the information from.** You are allowed to tick multiple options: | |
| Gynaecologist | General practitioner |
| Friend | Family |
| Media | Internet |
| Other | |
